# Lifestyle among long-term survivors of cancers in young adulthood

**DOI:** 10.1007/s00520-020-05445-6

**Published:** 2020-05-01

**Authors:** Synne-Kristin H. Bøhn, Hanne C. Lie, Kristin V. Reinertsen, Sophie D. Fosså, Hege S. Haugnes, Cecilie E. Kiserud, Jon Håvard Loge, Torbjørn Wisløff, Lene Thorsen

**Affiliations:** 1 Department of Oncology, National Advisory Unit on Late Effects after Cancer Treatment, Oslo University Hospital, Oslo, Norway; 2 Department of Behavioural Sciences in Medicine, Institute of Basic Medical Sciences, Faculty of Medicine, University of Oslo, Oslo, Norway; 3 Faculty of Medicine, University of Oslo, Oslo, Norway; 4 Department of Clinical Medicine, Arctic University of Tromsø, Tromsø, Norway; 5 Department of Oncology, University Hospital of North Norway, Tromsø, Norway; 6 Department of Oncology, Regional Advisory Unit in Palliative Care, Oslo University Hospital, Oslo, Norway; 7 Department of Community Medicine, Arctic University of Tromsø, Tromsø, Norway; 8 Department of Health Management and Health Economics, University of Oslo, Oslo, Norway; 9 Department of Clinical Service, Division of Cancer Medicine, Oslo University Hospital, Oslo, Norway

**Keywords:** Late effects, Unhealthy lifestyle, Physical activity, Overweight, Smoking

## Abstract

**Purpose:**

To investigate lifestyle in a population-based sample of long-term (≥ 5 years since diagnosis) young adult cancer survivors (YACSs), and explore factors associated with not meeting the lifestyle guidelines for physical activity (PA), body mass index (BMI), and smoking.

**Methods:**

YACSs (*n* = 3558) diagnosed with breast cancer (BC), colorectal cancer (CRC), non-Hodgkin lymphoma (NHL), acute lymphoblastic leukemia (ALL), or localized malignant melanoma (MM) between the ages of 19 and 39 years and treated between 1985 and 2009 were invited to complete a mailed questionnaire. Survivors of localized MM treated with limited skin surgery served as a reference group for treatment burden.

**Results:**

In total, 1488 YACSs responded (42%), and 1056 YACSs were evaluable and included in the present study (74% females, average age at survey 49 years, average 15 years since diagnosis). Forty-four percent did not meet PA guidelines, 50% reported BMI ≥ 25 and 20% smoked, with no statistically significant differences across diagnostic groups. Male gender, education ≤ 13 years, comorbidity, lymphedema, pain, chronic fatigue, and depressive symptoms were associated with not meeting single and/or an increasing number of lifestyle guidelines.

**Conclusion:**

A large proportion of long-term YACSs do not meet the lifestyle guidelines for PA, BMI, and/or smoking. Non-adherence to guidelines is associated with several late effects and/or comorbidities that should be considered when designing lifestyle interventions for YACSs.

**Electronic supplementary material:**

The online version of this article (10.1007/s00520-020-05445-6) contains supplementary material, which is available to authorized users.

## Introduction

Each year, approximately 130,000 individuals aged 20 to 39 years are diagnosed with cancer in Europe [1]. Improvements in detection and treatment have led to a relative 5-year survival rate of more than 80%, thus creating a rapidly growing population of long-term (≥ 5 years since diagnosis) young adult cancer survivors (YACSs) [2, 3]. Their life-saving treatment, however, places long-term YACSs at risk of late effects, such as fatigue, cardiovascular diseases, and second cancer [3–5].

Physical activity (PA), a healthy body mass index (BMI), and non-smoking are associated with a lower risk of cancer recurrence, morbidity, and mortality [6–8], and are considered key components to improve and preserve long-term health among cancer survivors [9]. Furthermore, healthy lifestyle behaviors (and conversely, unhealthy behaviors) are likely to cluster within individuals, e.g., those who are physically active are likely to not smoke [10]. Meeting several lifestyle guidelines provides superior health benefits compared with meeting only a single guideline [9]. Similar to the population in general, cancer survivors are therefore recommended to be physically active for at least 150 min with moderate intensity or 75 min with high intensity per week, maintain a healthy BMI, avoid smoking, and consume at least five daily servings of vegetables and fruits (“5-a-day”) [11, 12].

Despite the well-known health benefits of meeting these guidelines, a large proportion of cancer survivors are physically inactive, overweight and do not meet “5-a-day”, and few cancer survivors meet multiple lifestyle guidelines (7–40%) [10, 13]. To date, research on lifestyle in cancer survivors is predominantly based on populations diagnosed with cancer after the age of 50, examined less than 5 years since diagnosis [10, 13]. Although a cancer diagnosis may immediately motivate individuals to live a more healthy life [9], little is known about the lifestyle of those surviving 5 years and beyond.

The few studies which have investigated lifestyle in YACSs have also mostly included populations less than 5 years since diagnosis [14–16]. Two recent studies from the USA investigated lifestyle exclusively among long-term adolescent and YACSs, and found that 56–65% were not meeting the PA guidelines, and one in three were smoking [17, 18]. Generalizability of these US findings to European long-term YACS is, however, questionable due to differences in culture and health care systems.

For long-term YACSs, empirical knowledge on their lifestyle is lacking. To our knowledge, no previous studies have investigated the adherence to multiple lifestyle guidelines in long-term YACSs. Demographic characteristics, such as male gender, older age, and low education have been linked to unhealthy lifestyle behaviors among survivors diagnosed with cancer at a young age [19], but associations between lifestyle and cancer treatments and late effects, as well as other health characteristics, are scarcely explored in long-term YACSs. One might hypothesize that some groups of long-term YACSs might be more susceptible to an unhealthy lifestyle than others, e.g., a high treatment burden with subsequent late effects such as fatigue might limit individuals in meeting the PA guidelines. Knowledge on demographic, cancer-related, and health characteristics of those with an unhealthy lifestyle is required in order to identify subgroups that might need particular support, and to develop effective lifestyle interventions for long-term YACSs [15, 16].

On this background, the overall aim of the present study was to investigate lifestyle among long-term YACSs, based on data from a large population-based cross-sectional survey named The Norwegian childhood, adolescent, and young adult cancer survivor study (The NOR-CAYACS study) [20]. Specific aims were to:

1. Investigate the adherence to lifestyle guidelines among Norwegian long-term YACSs treated for breast cancer (BC), colorectal cancer (CRC), non-Hodgkin lymphoma (NHL), acute lymphoblastic leukemia (ALL), or localized malignant melanoma (MM).
2. Explore demographic, cancer-related, and health characteristics associated with not meeting single and an increasing number of guidelines for PA, BMI, and smoking.

Based on existing knowledge about lifestyle in other populations of cancer survivors, we hypothesized that most YACSs would not meet PA guidelines and/or be overweight, but that a minority would be smoking. Moreover, we hypothesized that low level of education, comorbid conditions, and late effects would be associated with not meeting lifestyle guidelines.

## Methods

### Design and study population

**Fig. 1:**
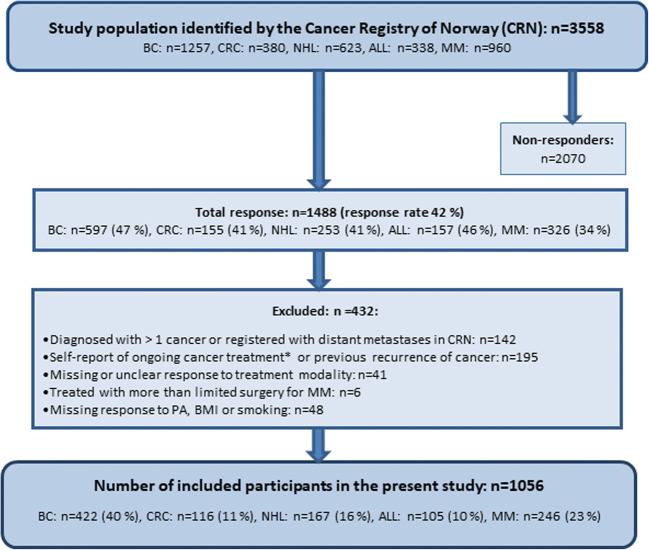
Flow chart of included participants. BC, breast cancer; CRC, colorectal cancer; NHL, non-Hodgkin lymphoma; ALL, acute lymphoblastic leukemia; MM, malignant melanoma. *BC survivors undergoing hormone therapy were retained in the sample (*n* = 22)

Details on study design and population have been described previously [20]. In brief, 3558 YACSs diagnosed with BC, CRC, NHL, or ALL, as well as a randomly selected subsample of MM, between the ages of 19 and 39 years during 1985–2009 were identified by the Cancer Registry of Norway (CRN), and invited to participate in a postal questionnaire-based survey. The selection of the cancer diagnoses was based on their relative frequent occurrence during young adulthood, on the good prognosis and the relatively high risk of late effects. YACSs of other relevant cancer types such as testicular cancer, Hodgkin lymphoma, and cervical cancer were not invited because survivors after these diagnoses already participated in other ongoing studies at our research unit at the time of survey. Exclusion criteria for the present study are described in Fig. 1.

### Variables and measurements

#### Lifestyle

*Physical inactivity* was defined as not meeting the guidelines of ≥ 150 min of moderate intensity PA or 75 min high intensity PA, or an equivalent combination of moderate and high intensity PA per week [11], using a modified version of the Godin Leisure Time Exercise Questionnaire (GLTEQ) [21]. The GLTEQ assesses the average frequency and number of minutes of mild, moderate, and vigorous leisure time PA during a typical week. The number of minutes within the different intensity levels of PA were calculated for each participant, and used to classify individuals as physically active (≥ 150 min of moderate intensity or ≥ 75 min of vigorous intensity per week) or inactive according to the PA guidelines.

*BMI* (kg/m^2^) was calculated from self-reported height and body weight, and categorized according to the World Health Organization’s categorization of BMI in adults, healthy weight (18.5–24.9 kg/m^2^), and overweight/obese (> 25.0–29.9 kg/m^2^) and obese (≥ 30 kg/m^2^) [22].

“*5*-*a*-*day*” was assessed by a question modified from the Nord-Trøndelag Health (HUNT) study [23], asking the participants how often they consume at least five daily servings of vegetables, fruits, and berries. Responses were categorized into meeting “5-a-day” (every day) and not meeting “5-a-day” (4–6 days per week/1–3 days per week/less than 1 day per week). Nutrition guidelines are complex, and for this paper, we chose to only include the measure on “5-a-day,” which has shown to be associated with other healthy eating habits [24]**.**

*Current smoking* was assessed by the question “Do you smoke?”, from the HUNT study [23]. Responses were dichotomized into yes (smoking daily or smoking now and then) versus no (discontinued smoking/never smoked).

*A more unhealthy lifestyle*: the number of lifestyle guidelines not met (physically inactive, BMI ≥ 25 and smoking) were summed for each participant (0 to 3). Because of the large proportion not meeting “5-a-day” (92%), “5-a-day” was not included in the score of a more unhealthy lifestyle.

#### Explanatory variables

Participants self-reported on demographic, cancer treatment, and health variables, while information on cancer type and initial stage was obtained from the CRN.

*Living with a partner* included marriage and cohabitation. *Education level* was dichotomized into ≤ 13 years (up to high school) versus > 13 years (college/university).

*Treatment intensity* was categorized as (1) limited surgery for localized MM (surgical removal of the skin lesion), (2) surgery and/or radiotherapy, (3) systemic treatment only, and (4) systemic treatment combined with surgery and/or radiotherapy.

*Number of comorbid conditions* was assessed using a modified version of the Charlson comorbidity index [25]. For each participant, the number of the following comorbid conditions ever experienced was summed and categorized as “no comorbidity,” “1–2 comorbid conditions,” and “> 2 comorbid conditions”: cardiovascular and pulmonary diseases, diabetes, kidney disease, gastro-intestinal disease, rheumatic disease, arthrosis, muscle/joint pain, epilepsy, and thyroid diseases.

*Presence of numbness in hands/feet* and *lymphedema* were categorized as yes/no. *Pain* was assessed by the pain item in the 12-Item Short Form Survey (SF-12) [26]. Responses were dichotomized as no (“not at all”/“a little bit”/“moderately”) versus yes (“quite a bit”/“extremely”). Using questions modified from the HUNT study [23], *trouble sleeping* was defined as experiencing one or more of the following three problems several times per week: “difficulties falling asleep at night,” “waking up repeatedly during the night,” and/or “waking up too early without being able to go back to sleep.”

*Depressive symptoms* were assessed using the nine-item Patient Health Questionnaire-9 (PHQ-9), which corresponds to the Diagnostic and Statistical Manual of Mental Disorders diagnostic criteria for major depressive disorders [27].The PHQ-9 contains 9 items. The frequency of experienced depressive symptoms during the last 2 weeks with response categories ranging from 0 (not at all) to 3 (nearly every day) is assessed. Increasing sum score (0 to 27) indicates higher level of depressive symptoms. *Anxiety symptoms* were measured by the seven-item anxiety subscale of The Hospital Anxiety and Depression Scale (HADS-A) [28], with response categories from 0 (not present) to 3 (highly present). An increasing sum score (0 to 21) indicates higher level of anxiety symptoms. Cronbach’s alphas were 0.87 for PHQ-9 and 0.83 for HADS-A in the present study population. HADS-A was used to assess level of anxiety symptoms.

*Chronic fatigue* was assessed by the Fatigue Questionnaire (FQ) [29]. FQ contains 11 items distributed on two subscales: physical fatigue (7 items) and mental fatigue (4 items). Each item is scored from 0 to 3, with increasing total score (0 to 33) implying higher levels of fatigue. To identify chronic fatigue, raw scores of each item were dichotomized (0 = 0, 1 = 0, 2 = 1, 3 = 1). Chronic fatigue was defined by a dichotomized sum score ≥ 4 and ≥ 6 months duration of fatigue [29]. Cronbach’s alphas for the present study population were 0.91 (physical subscale), 0.84 (mental subscale), and 0.92 (the whole scale).

### Statistical analyses

Continuous variables were described using mean and standard deviation (SD), and categorical variables were presented as numbers and percentages. Comparisons across diagnostic groups were performed with chi-square tests or one-way analysis of variance. Logistic regression analyses identified factors associated with not meeting single guidelines of PA, overweight, and smoking. Ordinal regression analyses were applied to identify factors associated with an increasing number of unhealthy lifestyle factors in terms of physical inactivity, overweight, and smoking (0 to 3), referred to as a more unhealthy lifestyle. Variables statistically significant associated with the dependent variable in unadjusted analyses (*p* < 0.05) were included as independent variables in the multivariable regression analyses. Limited surgery for localized MM was used as a reference group for treatment burden in the regression analyses.

All independent variables included in multivariable analyses were checked for multicollinearity, and all correlation coefficients were < > 0.8). Because of overlapping content in the items in FQ and PHQ-9, only chronic fatigue was included in multivariable analyses if both fatigue and depressive symptoms were statistically significant associated with the dependent variable in unadjusted analyses. For the ordinal regression analyses, the proportional odds assumption was confirmed by the test of parallel lines. Results from the multivariable analyses were presented as adjusted odds ratios (aOR) with 95% confidence intervals (95% CI). *P* values < 0.05 were considered statistically significant. Statistical analyses were performed using IBM SPSS statistics version 25.0.

### Compliance with ethical standards

The NOR-CAYACS study was approved by the South East Regional Committee for Medical and Health Research Ethics (no: 2015/232), the Norwegian Data Protection Authority (no: 15/00395-2/CGN), the Data Protection Officer at Oslo University Hospital and the CRN. Informed consent was obtained from all individual participants included in the study. The authors declare that they have no conflict of interest.

## Results

### Characteristics of participants

A total of 1488 (42%) YACSs responded. After exclusion of 432 responders, 1056 evaluable participants were retained (Fig. 1). Characteristics of evaluable responders versus non-responders are described in the online resource file.

Characteristics of the sample are presented in Table 1. In brief, 74% were female, 40% diagnosed with BC, 11% CRC, 16% NHL, 10% ALL, and 23% MM. Mean age at survey was 49.0 years (SD 7.7), and mean time since diagnosis was 15.2 years (SD 6.8). Forty-seven percent of the participants had received systemic treatment in combination with surgery and/or radiotherapy and 72% reported at least one comorbid condition.

**Table 1:** Demographic, cancer-related, and health characteristics of the participants

Variables	Total (*n* = 1056)
Demographic variables	
Female gender, *n* (%)	783 (74)
Age at survey, mean (SD)	49.0 (7.7)
Living with a partner^a^, *n* (%)	841 (80)
Living with children^b^, *n* (%)	415 (39)
Education > 13 years^c^, *n* (%)	624 (60)
Cancer-related variables	
Age at diagnosis, mean (SD)	32.8 (5.4)
Years since diagnosis, mean (SD)	15.2 (6.8)
Cancer type, *n* (%)	
Breast cancer	422 (40)
Colorectal cancer	116 (11)
Non-Hodgkin lymphoma	167 (16)
Acute lymphoblastic leukemia	105 (10)
Malignant melanoma	246 (23)
Treatment modality, *n* (%)	
Limited surgery for localized malignant melanoma	246 (23)
Surgery and/or radiotherapy	166 (16)
Systemic treatment alone	144 (14)
Systemic treatment combined with surgery and/or radiotherapy	500 (47)
Health variables	
Number of comorbid conditions, *n* (%)	
None	292 (28)
1–2	560 (53)
> 2	202 (19)
Numbness in hands/feet, *n* (%)	174 (18)
Lymphedema, *n* (%)	213 (22)
Pain^d^, *n* (%)	106 (10)
Trouble sleeping^e^, *n* (%)	469 (44)
PHQ-9 score^f^, mean (SD)	5.3 (4.8)
HADS-A score^g^, mean (SD)	4.7 (3.7)
Chronic fatigue^h^, *n* (%)	257 (25)

*SD*, standard deviation

Missing data are as follows: living with a partner/with children *n* = 2; education level *n* = 8; comorbid conditions *n* = 2; numbness in hands/feet *n* = 79; lymphedema *n* = 63; pain *n* = 9; trouble sleeping *n* = 1; PHQ-9 *n* = 5; HADS-A *n* = 4; chronic fatigue *n* = 16

^a^Married or cohabitant

^b^Aged < 18 years

^c^College/university

^d^Defined as pain interfering quite a bit or extremely with normal work

^e^Experiencing difficulties falling asleep at night, waking up repeatedly during the night, and/or waking up too early without being able to go back to sleep several times per week

^f^The Patient Health Questionnaire-9, range 0–27. Increasing score implies higher level of depressive symptoms

^g^The Hospital Anxiety and Depression Scale, anxiety subscale, range 0–21. Increasing score implies higher level of anxiety

^h^Elevated fatigue symptoms of at least 6 months duration

Percentages may not add up to 100 because of rounding

### Adherence to lifestyle guidelines

Among all YACSs, 44% were physically inactive, 50% were overweight, 20% were current smokers, and 92% did not consume “5-a-day” (Table 2). There were no statistically significant differences across the diagnostic groups (Table 2). Twenty-six percent met all three guidelines for PA, BMI, and smoking (Table 2).

**Table 2:** Adherence to lifestyle guidelines by cancer type

Variables	Total	BC	CRC	NHL	ALL	MM	*p* value
	*n* = 1056	*n* = 422	*n* = 116	*n* = 167	n = 105	*n* = 246	
Lifestyle variables, *n* (%)							
Physically inactive^a^	460 (44)	175 (42)	50 (43)	69 (41)	55 (52)	111 (45)	0.323
BMI ≥ 25	528 (50)	187 (44)	62 (53)	90 (54)	58 (55)	131 (53)	0.056
Current smoker	165 (20)	92 (22)	22 (19)	32 (19)	19 (18)	43 (18)	0.702
Not meeting 5-a-day^b^ (*n* = 1051)	744 (92)	381 (91)	106 (91)	156 (93)	101 (97)	221 (91)	0.226
Number of lifestyle guidelines met^c^							
0	57 (5)	22 (5)	3 (3)	10 (6)	13 (12)	9 (4)	
1	296 (28)	110 (26)	35 (30)	45 (26)	27 (26)	79 (32)	
2	433 (41)	168 (40)	55 (47)	71 (43)	39 (37)	100 (41)	
3	270 (26)	122 (29)	23 (20)	41 (25)	26 (25)	58 (24)	

*BC*, breast cancer; *CRC*, colorectal cancer; *NHL*, non-Hodgkin lymphoma; *ALL*, acute lymphoblastic leukemia; *BMI*, body mass index (kg/m^2^)

^a^Defined as not meeting physical activity guidelines of at least 150 min moderate exercise per week or 75 min of high-intensity exercise or an equivalent combination

^b^Defined as consuming at least five daily servings of fruits and vegetables

^c^Including guidelines for physical activity, BMI, and smoking

Percentages may not add up to 100 because of rounding

### Factors associated with not meeting lifestyle guidelines

Factors associated with physical inactivity, overweight, or smoking in unadjusted analyses are shown in Table 3.

**Table 3:** Characteristics of physically inactive, overweight, and currently smoking participants and factors associated with these behaviors

	Physical inactivity^a^						Overweight (BMI ≥ 25 kg/m^2^)						Current smoking (daily or now and then)					
		Unadjusted		Adjusted				Unadjusted		Adjusted				Unadjusted		Adjusted		
Variables		cOR	95% CI	aOR	95% CI	*p*		cOR	95% CI	aOR	95% CI	*p*		cOR	95% CI	aOR	95% CI	*p*
Socio-demographic variables																		
Sex, *n* (%)																		
Female (ref.)	340 (43)	1.0					348 (44)	1.0		1.0			163 (21)	1.0				
Male	120 (44)	1.02	0.77–1.35				180 (66)	*2.42*	1.82–3.22***	*2.50*	*1.80–3.45*	*< 0.001*	45 (17)	0.75	0.52–1.08			
Age at survey, mean (SD)	48.2 (8.0)	0.99	0.98–1.01				49.4 (7.5)	1.01	0.99–1.03				49.4 (7.0)	1.01	0.99–1.03			
Living with a partner, *n* (%)																		
Yes (ref.)	359 (43)	1.0					428 (51)	1.0					149 (18)	1.0		1.0		
No	101 (47)	1.21	0.90–1.64				99 (47)	0.84	0.62–1.13				58 (27)	*1.74*	1.23–2.47**	*1.50*	*1.02*–*2.21*	*0.041*
Living with children < 18 years, *n* (%)																		
Yes (ref)	169 (41)	1.0					192 (46)	1.0					69 (17)	1.0		1.0		
No	291 (46)	1.22	0.95–1.56				335 (52)	1.28	0.99–1.64				138 (22)	*1.38*	1.0–1.90*	1.10	0.78–1.54	0.604
Education level																		
> 13 years (ref.)	253 (41)	1.0		1.0			295 (47)	1.0		1.0			98 (16)	1.0		1.0		
≤ 13 years	203 (48)	*1.35*	1.05–1.73*	1.26	0.97–1.63	0.082	227 (54)	*1.29*	1.0–1.65*	1.14	0.87–1.49	0.353	108 (26)	*1.83*	1.35–2.49***	*1.63*	*1.18*–*2.27*	*0.003*
Cancer-related variables																		
Years since diagnosis, mean (SD)	15.5 (7.0)	1.01	0.99–1.03				15.5 (6.8)	1.01	0.99–1.03				15.4 (6.8)	1.01	0.98–1.03			
Treatment modality, *n* (%)																		
Reference^b^	111 (45)	1.0					131 (53)	1.0		1.0			43 (18)	1.0				
Surgery and/or radiotherapy	76 (46)	1.03	0.69–1.53				88 (53)	0.99	0.67–1.47	0.81	0.52–1.25	0.336	27 (16)	0.92	0.54–1.55			
Systemic treatment alone	72 (50)	1.22	0.81–1.84				83 (58)	1.19	0.79–1.81	0.88	0.56–1.40	0.599	30 (21)	1.24	0.74–2.09			
Systemic treatment with surgery and/or radiotherapy	201 (40)	0.82	0.60–1.11				226 (45)	*0.72*	0.53–0.98*	*0.62*	*0.44*–*0.89*	*0.009*	108 (22)	1.30	0.88–1.93			
Health variables, *n* (%)																		
Number of comorbid conditions																		
None (ref.)	116 (40)	1.0		1.0			131 (45)	1.0		1.0			53 (18)	1.0				
1–2	237 (42)	1.11	0.84–1.49	0.93	0.69–1.26	0.637	267 (48)	1.12	0.84–1.49	0.99	0.73–1.35	0.948	106 (19)	1.05	0.73–1.52			
> 2	105 (52)	*1.64*	1.14–2.36**	1.19	0.80–1.76	0.390	130 (64)	*2.22*	1.53–3.21***	*1.99*	*1.31*–*3.04*	*0.001*	48 (24)	1.41	0.91–2.18			
Numbness in hands/feet																		
No (ref.)	352 (44)	1.0					398 (50)	1.0					151 (19)	1.0				
Yes	81 (47)	1.12	0.80–1.55				83 (48)	0.93	0.67–1.29				37 (21)	1.17	0.78–1.75			
Lymphedema																		
No (ref.)	342 (44)	1.0					372 (48)	1.0		1.0			135 (17)	1.0		1.0		
Yes	94 (44)	1.01	0.75–1.37				*121 (57)*	*1.44*	1.06–1.96*	*1.77*	*1.25*–*2.50*	*0.001*	57 (27)	*1.75*	1.22–2.49**	*1.67*	*1.15*–*2.41*	*0.007*
Pain interfering with normal work																		
No (ref.)	393 (42)	1.0		1.0			460 (49)	1.0		1.0			177 (19)	1.0				
Yes	61 (58)	*1.89*	1.26–2.84**	1.39	0.89–2.16	0.151	63 (59)	*1.53*	1.02–2.30*	1.13	0.69–1.83	0.633	28 (26)	1.55	0.98–2.46			
Trouble sleeping																		
No (ref.)	233 (40)	1.0		1.0			288 (49)	1.0					106 (18)	1.0				
Yes	227 (48)	*1.42*	1.11–1.82**	1.24	0.95–1.61	0.114	240 (51)	1.08	0.85–1.38				102 (22)	1.26	0.93–1.71			
PHQ-9 score^c^, mean (SD)	6.1 (5.1)	*1.07*	1.04–1.10*** ^*f*^				5.7 (4.9)	*1.04*	1.01–1.06**	*1.03*	*1.01*–*1.06*	*0.042*	6.1 (5.0)	*1.04*	1.01–1.07**	1.0	0.96–1.05	0.941
HADS-A score^d^, mean (SD)	4.8 (3.8)	1.02	0.99–1.06				4.7 (3.8)	1.01	0.98–1.04				5.3 (3.8)	*1.05*	1.01–1.10*	1.04	0.98–1.10	0.236
Chronic fatigue^e^, *n* (%)																		
No (ref.)	314 (40)	1.0		1.0			393 (50)	1.0					149 (19)	1.0				
Yes	138 (54)	*1.73*	1.30–2.30***	*1.50*	*1.11*–*2.03*	*0.009*	130 (51)	1.02	0.77–1.35				53 (21)	1.11	0.78–1.57			

*BMI*, body mass index; *95% CI*, 95% confidence interval; *SD*, standard deviation; *cOR*, crude odds ratio; *aOR*, adjusted odds ratio; *Ref*., reference

Italics**:** statistically significant result (*p* < 0.05)

Numbers included in adjusted multivariable analyses were 1024 for physically inactive, 981 for overweight, and 985 for smoking

^a^Physical inactivity was defined as not meeting physical activity guidelines of at least 150 min moderate or 75 min vigorous physical activity per week

^b^Limited skin surgery for malignant melanoma

^c^The Patient Health Questionnaire-9, range 0–27. Increasing score implies higher level of depressive symptoms

^d^The Hospital Anxiety and Depression Scale, anxiety subscale, range 0–21. Increasing score implies higher level of anxiety

^e^Defined as elevated level of fatigue of at least 6 months duration

^f^Not included in multivariable analyses due to overlap with chronic fatigue

*p < 0.05

**p < 0.01

***p < 0.001

In multivariable analyses, only chronic fatigue remained associated with physical inactivity (aOR 1.50, 95% CI 1.11–2.03) (Table 3). Male gender (aOR 2.50, 95% CI 1.80–3.45), > 2 comorbid conditions (aOR 1.99, 95% CI 1.31–3.04), lymphedema (aOR 1.77, 95% CI 1.25–2.50), and increasing levels of depressive symptoms (aOR 1.03, 95% CI 1.01–1.06) were associated with being overweight. Systemic treatment combined with surgery and/or radiotherapy was negatively associated with overweight (aOR 62, 95% CI 0.44–0.89). Living without a partner (aOR 1.50, 95% CI 1.02–2.21), education ≤ 13 years (aOR 1.63, 95% CI 1.18–2.27) and lymphedema (aOR 1.67, 95% CI 1.15–2.41) were positively associated with smoking (Table 3).

Factors associated with a more unhealthy lifestyle in unadjusted analyses are shown in Table 4. Male gender (aOR 1.80, 95% CI 1.37–2.37), education ≤ 13 years (aOR 1.44, 95% CI 1.13–1.84), > 2 comorbid conditions (aOR 1.57, 95% CI 1.08–2.29), lymphedema (aOR 1.37, 95% CI 1.02–1.84), and pain (aOR 1.54, 95% CI 1.0–2.35) were associated with a more unhealthy lifestyle in multivariable ordinal regression analyses.

**Table 4:** Ordinal logistic regression analyses of potential associated factors with not meeting multiple lifestyle guidelines*

	Unadjusted			Adjusted**		
	cOR	95% CI	*p*	aOR	95% CI	*p*
Sex, *n* (%)						
Female (ref.)	1.0			1.0		
Male	*1.46*	*1.14–1.88*	*0.003*	*1.80*	*1.37–2.37*	*< 0.001*
Age at survey, mean (SD)	1.01	0.99–1.02	0.306			
Living with a partner, *n* (%)						
Yes (ref.)	1.0					
No	1.26	0.96–1.66	0.100			
Living with children < 18 years, *n* (%)						
Yes (ref)	1.0			1.0		
No	*1.43*	*1.14–1.80*	*0.002*	1.21	0.94–1.54	0.137
Education level, *n* (%)						
> 13 years (ref.)	1.0			1.0		
≤ 13 years	*1.65*	*1.31–2.07*	*< 0.001*	*1.44*	*1.13–1.84*	*0.003*
Cancer-related variables						
Years since diagnosis, mean (SD)	1.01	0.99–1.03	0.110			
Treatment modality, *n* (%)						
Reference^a^	1.0					
Surgery and/or radiotherapy	0.99	0.70–1.43	0.987			
Systemic treatment alone	1.24	0.85–1.80	0.269			
Systemic treatment with surgery and/or radiotherapy	0.80	0.61–1.06	0.120			
Health variables						
Number of comorbid conditions, *n* (%)						
None (ref.)	1.0			1.0		
1–2	1.11	0.86–1.44	0.435	0.94	0.71–1.24	0.641
> 2	*2.17*	*1.16–3.03*	*< 0.001*	*1.57*	*1.08–2.29*	*0.018*
Numbness in hands/feet, *n* (%)						
No (ref.)	1.0					
Yes	1.05	0.78–1.41	0.764			
Lymphedema, *n* (%)						
No (ref.)	1.0			1.0		
Yes	*1.46*	*1.10–1.93*	*0.008*	*1.37*	*1.02–1.84*	*0.037*
Pain interfering with normal work, *n* (%)						
No (ref.)	1.0			1.0		
Yes	*2.10*	*1.45–3.05*	*< 0.001*	*1.54*	*1.0–2.35*	*0.048*
Trouble sleeping, *n* (%)						
No (ref.)	1.0			1.0		
Yes	*1.35*	*1.08–1.68*	*0.009*	1.10	0.86–1.42	0.450
PHQ-9 score^b^, mean (SD)	*1.07*	*1.05–1.10*	*< 0.001*^*c*^			
HADS-A score^d^, mean (SD)	*1.03*	*1.0–1.07*	*0.026*	1.02	0.98–1.05	0.357
Chronic fatigue^e^, *n* (%)						
No (ref.)	1.0			1.0		
Yes	*1.38*	*1.06–1.79*	*0.015*	1.09	0.81–1.46	0.573

95% CI = 95% confidence interval; *SD*, standard deviation; *cOR*, crude odds ratio; *aOR*, adjusted odds ratio; *Ref*., reference. Variables associated (*p* < 0.05) (italics**)** with not meeting an increasing number of guidelines in unadjusted analyses were included as explanatory variables in the adjusted analyses

*Not meeting an increasing number of PA, BMI, and/or smoking guidelines

**Numbers included in multivariable analyses were 968

^a^Limited surgery for malignant melanoma

^b^The Patient Health Questionnaire-9

^c^Not included in multivariable analyses due to overlap with chronic fatigue

^d^The Hospital Anxiety and Depression Scale, anxiety subscale

^e^Elevated fatigue symptoms of at least 6 months duration

## Discussion

This large population-based study on lifestyle among long-term YACSs shows that the majority of long-term YACSs are physically inactive, overweight, and/or not meeting “5-a-day,” and that one in five are smokers. Only one in four YACSs meet the combination of PA, BMI, and smoking guidelines. Non-adherence to lifestyle guidelines is associated with male gender, living without a partner, education ≤ 13 years, comorbid conditions, lymphedema, pain, increasing levels of depressive symptoms, and/or chronic fatigue.

Importantly, the diversity of measures, population characteristics, and cultural differences across studies limit direct comparison of our findings with previous results on lifestyle among cancer survivors. Taking this into account, long-term YACSs in our study seemed to be overall equally or more adherent to lifestyle guidelines than cancer survivors in general [10, 13, 15, 17, 19]. Compared with our finding that 44% of YACSs are physically inactive, Warner et al. reported physical inactivity in 56–65% of US long-term adolescent and YACSs [17]. Also, the proportion not meeting PA guidelines in our study is lower than findings among survivors diagnosed with cancer at an older age (50–75%) [10, 13, 30]. In agreement with our findings, and using the same PA questionnaire, Bélanger et al. [15] found that 48% were physically inactive among Canadian YACSs of various cancer types diagnosed between the ages of 20 to 44 years. However, most of these participants were not long-term survivors (i.e., < 5 years since diagnosis).

The prevalence of overweight in our study (50%) is also in agreement with findings in Bélanger et al.’s study (53%) [15], and with findings in US survivors of BC and CRC diagnosed before the age of 50 and examined almost 10 years after diagnosis (55%) [31]. Higher proportions of overweight have been found among survivors diagnosed with cancer further into adulthood (60–75%) [13, 30]. The proportion of 20% smokers in our study was lower than reported among female adolescent survivors and YACSs in US studies (≈ 30%) [17, 18], but higher than found among older adult cancer survivors and the YACSs in the study by Bélanger et al. (13%) [13, 15].

Our results are also similar to the self-reported prevalence of overweight (48%) and smoking (women 17%, men 22%) in the general Norwegian general population, while the proportion of physically inactive individuals in the general population (33%) is somewhat lower than among the YACSs (44%) [32, 33].

Furthermore, 92% of the participants in our study did not meet “5-a-day,” which is congruent with findings among the adolescent and YACSs in the study by Warner et al. [17] (up to 89% not meeting “5-a-day”) and the general Norwegian population (86%) [34]. In other populations of cancer survivors, somewhat higher proportions of survivors eating “5-a-day” have been reported (30–45%) [31, 35]. Given that close to all participants in our sample were not meeting “5-a-day,” we chose to not explore associated factors. A broader exploration of nutrition, e.g., guidelines on red meat, fish, sodium, and added sugar, would probably provide more information about the characteristics of long-term YACSs not meeting nutrition guidelines.

Assuming that long-term YACSs are aware of their risk for late effects following treatment, one could expect that they would be more motivated for having a healthy lifestyle than the general population. Due to their low treatment burden, one might hypothesize that survivors of localized MM would be more comparable to the population in general than to YACSs with a higher cancer treatment burden. However, adherence to lifestyle guidelines did not differ across the diagnostic groups in our study.

In sum, our findings suggest that despite their increased risk of a poorer health, long-term YACSs do not seem more likely of having a healthy lifestyle than the general population. One explanation for this might be lack of knowledge about the importance of a healthy lifestyle and their risk of late effects. In Norway, systemic follow-up programs including information on lifestyle issues for cancer survivors are lacking. Previous research has demonstrated limited knowledge about late effects among both cancer survivors [36] and general practitioners (GPs) [37]. Furthermore, in a recent systematic review, Tollosa et al. found that survivors 5 years or less from diagnosis had better health behavior than long-term survivors [13], suggesting that it is challenging to maintain a healthy lifestyle after cancer as time goes by. Moreover, as some late effects appear several years after treatment, cancer survivors might not be motivated for a healthy lifestyle until potential health problems occur. To the contrary, poor health and late effects after cancer may also limit the ability to obtain or maintain a healthy lifestyle [38].

Lifestyle interventions in cancer survivors must be targeted towards their unique needs and challenges [9]. We found that chronic fatigue was associated with not meeting PA guidelines, which is in line with previous research on fatigue and PA in survivors of lymphoma [39], CRC [40] and BC [41]. Fatigue is also one of the most commonly reported barriers for PA among cancer survivors in general [38]. PA is, however, also recommended to improve fatigue among cancer survivors, as physical inactivity and subsequent loss of muscle mass and physical function may worsen fatigue symptoms [42].

Also in agreement with previous findings among cancer survivors in general, being overweight in the present study was associated with male gender [30], comorbid conditions [39], and depressive symptoms [19]. We found that long-term YACSs who had received multimodal therapy were less likely to be overweight than MM survivors treated with limited surgery. This is in line with the findings in a recent study by our group reporting that receipt of three or more treatment regimens was associated with a decreased risk of being overweight in long-term lymphoma survivors treated with high-dose chemotherapy with autologous stem cell support [39]. However, research in BC survivors has reported large variations in weight change (gain, maintenance, and loss) during and after adjuvant systemic treatments [43].

The finding that only one in four long-term YACSs met all guidelines with regard to PA, BMI, and non-smoking is comparable with the results in the study by Spector et al., showing that 20% of older long-term NHL survivors met these three guidelines [35]. Also congruent with our findings, Tollosa et al. estimated that 23% of adult cancer survivors met a combination of several lifestyle recommendations [13]. Considering their long life-expectancy with risk of late effects and future health challenges associated with aging, adhering to a combination of multiple lifestyle guidelines might be particularly important for YACSs.

Our findings indicate a need to inform YACSs and health personnel involved in the follow-up of YACSs about the benefits of a healthy lifestyle also as a preventive measure against late effects. Such information may be conveyed through courses for cancer survivors and health personnel involved in the follow-up care of cancer survivors, and by establishing guidelines for lifestyle advice as part of follow-up. Moreover, focus on lifestyle and long-term health should be implemented in individual care plans and patient information (brochures/electronically). Patients should receive information or counseling about the benefits of a healthy lifestyle in a manner tailored to their needs and health literacy levels.

The main strength of this study is the large national population-based sample of YACSs, which is an understudied population in terms of long-term cancer survivorship [44]. Our study contributes with new knowledge about lifestyle and its associations to late effects, assessed with established patient-reported outcome measures. Such measures are essential to capture patient perspectives and symptoms that are subjective in nature and may lack universal diagnostic criteria (e.g., fatigue) [45]. Limitations include the cross-sectional design precluding causal conclusions, and the reliance on self-reported treatment data. The response rate of 42% and the high proportion of females and BC survivors might increase the risk of bias. However, Lie et al. recently found low risk of non-response bias in the NOR-CAYACS study on a wide range of survey outcomes, including lifestyle [20].

## Conclusion

Many long-term YACSs are not meeting one or more of the public guidelines for PA, BMI, and smoking. Health personnel involved in the follow-up of YACSs must have knowledge and focus on late effects and healthy lifestyle behaviors. YACSs with male gender, who are living without a partner, with education ≤ 13 years, comorbid conditions, lymphedema, pain, increasing levels of depressive symptoms, and/or chronic fatigue might have an increased risk of not meeting one or more of these guidelines. YACSs with these characteristics might need special attention to achieve and maintain a healthy lifestyle.

## Electronic supplementary material

ESM 1 (DOCX 14 kb)

## Data Availability

The authors have full control of all primary data and the journal may review the data if requested.
