# Supplementary material for: Lifestyle among long-term survivors of cancers in young adulthood
Source: Support Care Cancer. 2020 May 1;29(1):289–300. doi: 10.1007/s00520-020-05445-6 (PMC7686209; doi:10.1007/s00520-020-05445-6)
Supplement: Supplementary file 1 — (DOCX 14 kb) [file 520_2020_5445_MOESM1_ESM.docx]

Supplementary material: characteristics of non-responders versus responders

|  | Non-responders (n=1838)* | Included responders (n=1056) | P-value |
| --- | --- | --- | --- |
| Sex, (n, %) |  |  |  |
| Female | 1279 (70) | 783 (74) | .009 |
| Male | 559 (30) | 273 (26) |  |
| Age at survey, mean (SD) | 48 (8) | 49 (8) | .001 |
| Age at diagnosis, mean (SD) | 33 (5) | 33 (5) | .476 |
| Time since diagnosis, mean (SD) | 14 (7) | 15 (7) | .001 |
| Cancer type, n (%) |  |  |  |
| BC | 563 (31) | 422 (40) | <.001 |
| CRC | 184 (10) | 116 (11) |  |
| NHL | 342 (19) | 167 (16) |  |
| ALL | 178 (10) | 105 (10) |  |
| MM | 571 (31) | 246 (23) |  |

Abbreviations: BC=breast cancer. CRC=colorectal cancer. NHL=non-Hodgkin lymphoma. ALL=acute lymphoblastic leukemia. MM=malignant melanoma.

*Non-responders with >1 cancer diagnosis, recurrence or distant metastasis excluded from non-responders (n=232).
